# Wave Propagation Analysis in the Homogenized Second-Gradient Medium: A Direct and Inverse Approach

**DOI:** 10.3390/ma18184248

**Published:** 2025-09-10

**Authors:** Fadheelah Al Fayadh, Hassan Lakiss, Hilal Reda

**Affiliations:** 1 Faculty of Engineering, Section III, Lebanese University, Campus Rafic Hariri, Beirut 1001, Lebanon; fadeelafaisal282@gmail.com; 2 Faculty of Engineering, Islamic University of Lebanon (IUL), Beirut 1001, Lebanon; pres.cal@iul.edu.lb; 3 Computation-Based Science and Technology Research Center, The Cyprus Institute, 2121 Nicosia, Cyprus

**Keywords:** homogenization, variational principles, wave propagation, composite materials, microstructure

## Abstract

In this work, we develop a method for homogenizing effective second-order gradient continuum models for 2D periodic composite materials. A constitutive law is formulated using a variational approach combined with the Hill macro-homogeneity condition for strain energy. Incorporating strain gradient effects enhances the constitutive law by linking the hyperstress tensor to the second-order gradient of displacement, capturing elastic size and microstructure effects beyond classical Cauchy elasticity. The effective strain gradient moduli are calculated for composites exhibiting strong internal length effects, validating the proposed approach by computing the strain energy at different scales. Additionally, we develop an inverse homogenization method to compute local mechanical properties (properties of the constituents) given known global properties (effective properties), showing good agreement with the literature data. This framework is extended to study wave propagation by analyzing longitudinal and shear waves in 2D composite materials. The effects of inclusion shape and volume percentage on wave propagation are examined, revealing that elliptic inclusions lead to a slight increase in both modes of propagation. Finally, we investigate the impact of property contrast between the inclusion and matrix, demonstrating its influence on wave dispersion.

## 1. Introduction

Composite materials are a group of materials that are increasingly being used in industrial technology due to their advantageous mechanical properties. The continuous development of manufacturing processes and the expanding applicability of these materials in many branches of engineering, such as automobile, devices, and energy storage, has resulted in a need to develop new methodologies for determining their effective mechanical, thermal, and electrical properties. Many micromechanical models created for the prediction of the effective mechanical properties of materials concentrate on elastic moduli, such as the Voight and Reus models [1,2,3].

A detailed analysis of the impacts of homogenization methods on the estimation of the effective mechanical properties of composites can be found in Gan et al. [4]. The homogenization results were compared with the Voigt/Reuss approximation [2,3]. Additionally, homogenization methods for Cauchy-type media, as outlined by Truesdell and Noll [5], consider only displacement as a degree of freedom (taking the first gradient of displacement in the constitutive law into account) while neglecting higher-order (rotation) and higher-gradient deformations, limiting their ability to describe certain significant mechanical and physical phenomena [6,7], especially the dispersive behavior of the medium.

At microscopic scales, where the heterogeneous nature of materials causes size effects due to the contrast between properties (ratio of properties between the constituents) and non-local behavior, the limitations of a Cauchy continuum in fully understanding the behaviors of the materials becomes an issue. Classical homogenization procedures used for Cauchy-type media fail when the wavelength of an external loading, incident wave, or deformation exceeds the microstructure’s size. The absence of the length scale parameter in the classical continuum elasticity theory of Cauchy types leads to an inaccurate description of structural behaviors and phase velocities of wave propagation [8]. To address these limitations, homogenization towards generalized effective continua that extends the classical Cauchy framework, introducing scale-dependent effects and improving its validity beyond the size effect separation assumption, must be discussed [9,10].

By introducing spatial interactions at the microlevel and characteristic length scales, enhanced continuum formulations can incorporate the microstructural scale to account for the influence of nearby points in constitutive equations. Micropolar media and strain gradient continua are extensions of the Cauchy classical notion of a continuous medium and can be divided into two categories. Higher-order continuum theories have been developed to capture microstructural behavior and size-dependent effects that Cauchy classical elasticity fails to describe. These models introduce additional degrees of freedom and higher-order kinematic descriptors to better represent the mechanical behavior of composite materials.

One of the models is the Cosserat medium, introduced by the Cosserat brothers [11], in which independent rotations are introduced. In this framework, local rotations are treated as separate kinematic fields in addition to the displacement field. This allows the model to capture behaviors such as couple stresses and rotational inertia, which are particularly relevant in granular media, foams, or materials with pronounced microstructure.

Building upon this concept, the micromorphic medium developed by Eringen, Suhubi and Mindlin [9,10], and Germain [12] extends the Cosserat theory by allowing each material point to possess an independent microdeformation tensor. This tensor can account not only for local rotations but also for local stretch and shear within the microstructure. As a result, micromorphic media can describe more complex internal behaviors such as the deformation of inclusions, metamaterials, and lattice structures.

**Figure 1:**
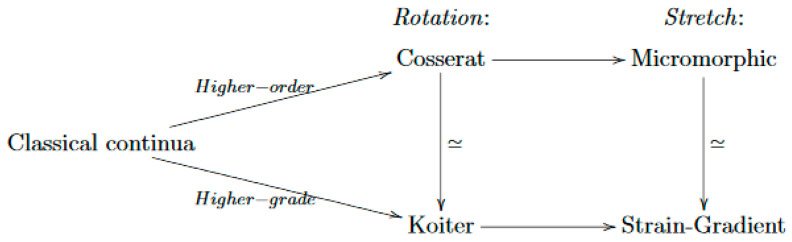
Schematic of classical continua.

In contrast, higher-gradient continua such as the Koiter medium and the second-gradient medium do not introduce new degrees of freedom. Instead, they enrich the Cauchy classical theory by incorporating higher-order spatial gradients of the displacement field. For example, the Koiter medium, rooted in the asymptotic expansion of three-dimensional elasticity for thin structures (plates and shells), includes both displacements and rotations derived from the geometry of the mid-surface. Meanwhile, the second-gradient medium [13] introduces the gradient of strain or the second gradient of displacement into the strain energy formulation. This allows the theory to capture size effects and strain localization by introducing internal length scales into the model, which are essential in the analysis of materials at small scales, such as in nano-mechanics and fracture mechanics (Figure 1). While maintaining the same degrees of freedom as classical continua, the strain energy density function in higher-gradient continua takes the gradient of deformation as its arguments.

Remarkably, as the number of microstructural unit cell periods rises, the effects of strain gradients asymptotically converge. Recently, Monchiet et al. [14] used quadratic boundary conditions applied on a composite to capture the local strain gradient effect, while maintaining the effective Cauchy-type medium. The asymptotic homogenization approach avoids the uncertainties associated with the classical homogenization because the source term in the second-order unit cell problem vanishes for a homogeneous medium [14,15]. Ayad et al. [16] evaluated wave propagation in a periodic 1D laminate framework using a second-gradient macroscopic constitutive model that was generated from Hamiltonian-based formulations and the internal strain gradient energy. Additionally, Ayad et al. [16] used a higher-order dynamic homogenization method to examine how micro-inertia and higher-order contributions affect the mechanics of composite structures. In order to provide a more realistic description of the dynamic behavior of composite beams in a 1D setting, this technique integrates strain gradient terms and micro-inertia effects.

The effects of microstructure—particularly the contrast between inclusion and matrix properties—on mechanical wave propagation within a second-gradient medium were studied by Forest et al. [13]. The authors highlighted the influence of the deformation gradient on wave behavior. Similarly, Berezovski et al. [17] introduced rotation as an additional degree of freedom and investigated wave propagation in a micromorphic medium. Their findings demonstrated that, in addition to the traditional longitudinal and shear modes, an additional rotational mode emerges due to the micromorphic nature of the effective medium. The authors in [18,19] investigated wave propagation in a generalized medium and took the gradient of deformation and the rotation as additional degrees of freedom. They emphasized the influence of microstructure on dispersion characteristics and the emergence of additional wave modes. Iliopoulos et al. [20] extended this analysis by studying the impacts of material heterogeneity on the phase velocity of the wave and attenuation. Réthoré et al. [21] focused on numerical approaches for modeling wave propagation in complex heterogeneous media, incorporating gradient effects to capture localized strain phenomena and discontinuities (presence of defects) more accurately. Consequently, these studies highlight the role of enriched (second-gradient and micromorphic) continuum theories in describing wave dispersion and microstructural effects. It is worth noting that, while finite element methods may exhibit numerical dispersion and challenges in resolving high-frequency modes in elastodynamic problems, the Boundary Element Method (BEM) has recently emerged as a powerful alternative, particularly in the frequency domain, as it naturally satisfies radiation conditions and reduces the dimensionality of the problem discussed in [21].

This study develops an effective second-order gradient continuum model for 2D composite materials, incorporating a variational approach and the Hill macrohomogeneity condition to compute mechanical properties. Section 2 introduces the classical first-gradient variational homogenization for Cauchy-type media, providing the foundation for effective property estimation. In Section 3, we develop an inverse homogenization approach to compute local material properties and validate it using literature data. Section 4 extends the classical variational approach to strain gradient homogenization, formulating a generalized medium and analyzing the influence of inclusion shape and volume percentage on the global effective properties. More specifically, we compare the effects of spherical and elliptical inclusions, showing their impact on composite behavior. Finally, Section 5 examines wave propagation in composite materials, emphasizing the role of strain gradient effects and property contrast on longitudinal and shear modes. We highlight how second-order effects influence wave dispersion and natural frequency shifts, providing insights into composite material design.

## 2. Cauchy-Type Medium: First-Gradient Variational Homogenization

The Hill–Mandel lemma [22] for macrohomogeneous Cauchy-type materials states that the elastic energy of the effective continuum at the macroscopic level is equivalent to the average microscopic energy assessed over the unit cell:

(1) $$W_{M}\left(E\right)=\frac{1}{2}E:C^{hom}:E={\langle w_{\mu }\left(\varepsilon \left(y\right)\right)\rangle }_{Y}={\langle \frac{1}{2}\varepsilon \left(y\right):C\left(y\right):\varepsilon \left(y\right)\rangle }_{Y}$$

in which $C\left(y\right)$ is the microscopic rigidities (fourth-order tensor), $C^{hom}$ is the effective (macroscopic) rigidity of the composite materials; $W_{M}\left(E\right)$ denotes the density macroscopic strain energy of the effective homogeneous Cauchy-type medium; wμ denotes the local strain energy density of the individual components (matrix or filler), ε is the local deformation and the bracket represents the volume averaging over the unit cell Y, viz ${\langle \left(.\right)\rangle }_{Y}:=\frac{1}{\left|Y\right|}\int_{Y}\left(.\right)dV_{y}$.

**Figure 2:**
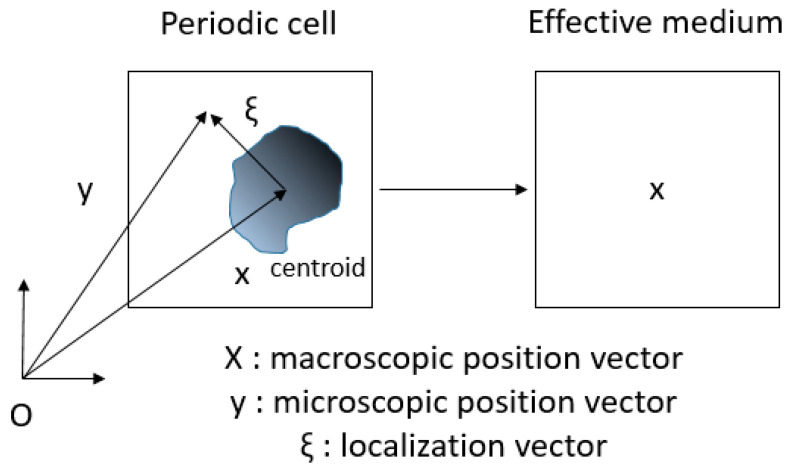
Composite domain (**left**) in the unit cell Y and the homogeneous substitution medium (**right**).

Three different scales can be distinguished: the macroscopic structural scale, the intermediate mesoscopic scale, and the microscopic structural scale (micrometers scale). The microscopic position is indicated by y (Figure 2), while the macroscopic position is represented by x. The mesoscopic strain is implicitly dependent on the macroscopic position, meaning that **E** = **E**(**x**). In order to differentiate between the two scales, mesoscopic and macroscopic fields are expressed with capital letters, whereas microscopic variables are represented with lowercase letters. In order to set the stage, the deformation vector is decomposed additively into a homogeneous part, $\varepsilon^{hom}\left(y\right)$, affine in the macrostrain and corresponding to the effective Cauchy continuum and a periodic fluctuation, denoted as $\tilde{\varepsilon }(y)\in H_{per}^{1}(Y)$ for the Sobolev space of a Y-periodic deformation:

(2) $$\begin{array}{c} \varepsilon \left(y\right)=\varepsilon^{hom}\left(x\right)+\tilde{\varepsilon }\left(y\right)\\ \varepsilon^{hom}\left(x\right)=u^{hom}\left(x\right){⊗}^{S}\nabla_{y}=E \end{array}$$

The homogeneous displacement $u^{hom}$ explains the effect of an “effective” isotropic continuum, which corresponds to and is analogous to a strain medium. This means that the effective isotropic medium is perturbed, $\tilde{u}(y)$, to the position of a macroscopic movement, which is defined by the homogeneous deformation $E\left(x\right)$. All displacements of the strain energy density, out of all possible spherical displacements, are usually accepted as the minimum energy strain density:

(3) $$W_{M}\left(E\right)=\underset{\tilde{u}\in H_{per}^{1}(Y)}{Min}\left\{\int_{Y}\frac{1}{2}C\left(y\right):\left(E+\tilde{u}(y)⊗\nabla_{y}\right):\left(E+\tilde{u}(y)⊗\nabla_{y}\right)dV_{y}\right\}$$

Taking the balance of linear momentum at the microlevel in the absence of body forces and its dyadic moment with the relative microscopic position into consideration and describing the self-equilibrium of the unit cell leads to the following:

(4) $$\begin{array}{c} \sigma \left(y\right)=\frac{\partial W_{\mu }\left(\varepsilon \right)}{\partial \varepsilon \left(y\right)}\\ \forall y\in Y,\;{div}_{y}\sigma \left(y\right)=0\\ -div_{y}\left\{C\left(y\right):\left(E+\tilde{u}(y)⊗\nabla_{y}\right)\right\}=0\\ \tilde{u}\in H_{per}^{1}(Y) \end{array}$$

where $\sigma \left(y\right)$ is the local stress tensor. because BVP (4) is linear in the mesoscopic strain E(x), the periodic fluctuation can be linearly stated using displacement localizators, ensuring that it holds (up to a function of x, which is irrelevant for further effective moduli calculations and can therefore be ignored).

(5) $$\tilde{u}(y)=H^{E}(y)\cdot E\left(x\right)$$

The third-order displacement localizator, $H^{E}\left(y\right)$, is periodic and dependent on the microscopic position variable. The following BVP is satisfied by the localizators when expression (5) is reinserted into BVP (4) and the knowledge that the macrostrain is independent of the microscopic variable is used:

(6) $$\left|\begin{array}{c} -div_{y}\left\{C\left(y\right):\left(I_{4}+H^{E}(y)⊗\nabla_{y}\right)\right\}=0\\ H^{E}(y)\;Y-periodic \end{array}\right.$$

with $I_{4}$ being the fourth-order identity tensor therein.

Next, using the mesoscopic strain-based potential, the homogenized first-gradient rigidity tensor is assessed: the partial derivative of the average Cauchy stress over the unit cell Y is elaborated upon.

(7) $$\Sigma :=\frac{\partial W_{M}\left(E\right)}{\partial E}\equiv C^{hom}:E$$

where Σ is the global stress tensor. The last relation implicitly defines the fourth-order tensor of effective rigidities, $C^{hom}$. Furthermore, because the function specified on the right-hand side of relation (3) is regular for macrostrain **E**, which acts as a parameter in the integrand, partial derivatives and integral can be permuted, such that the following holds:

(8) $$\begin{array}{c} \Sigma :=\frac{\partial W_{M}\left(E\right)}{\partial E}=\underset{\tilde{u}\in H_{per}^{1}(Y)}{Min}\frac{\partial }{\partial E}\left\{\int_{Y}\frac{1}{2}C\left(y\right):\left(E+\nabla_{y}\tilde{u}(y)\right):\left(E+\tilde{u}(y)⊗\nabla_{y}\right)dV_{y}\right\}=\\ =\left\{\int_{Y}C\left(y\right):\left(I_{4}+H^{E}(y)⊗\nabla_{y}\right)dV_{y}\right\}:E \end{array}$$

To obtain the final relation, the decomposition in (5) is used. A comparison of relations (7) and (8) leads to the classical statement of the homogenized tensor for the effective Cauchy continuum:

(9) $$C^{hom}=\left\{\int_{Y}C\left(y\right):\left(I_{4}+H^{E}(y)⊗\nabla_{y}\right)dV_{y}\right\}$$

Note that the term $\left(I_{4}+\nabla_{y}H^{E}(y)⊗\nabla_{y}\right)$ in (9) represents the strain localizator. The same effective tensor is obtained using double-scale asymptotic expansion methods [22].

## 3. Determination of Local Properties Using Inverse Homogenization

**Figure 3:**
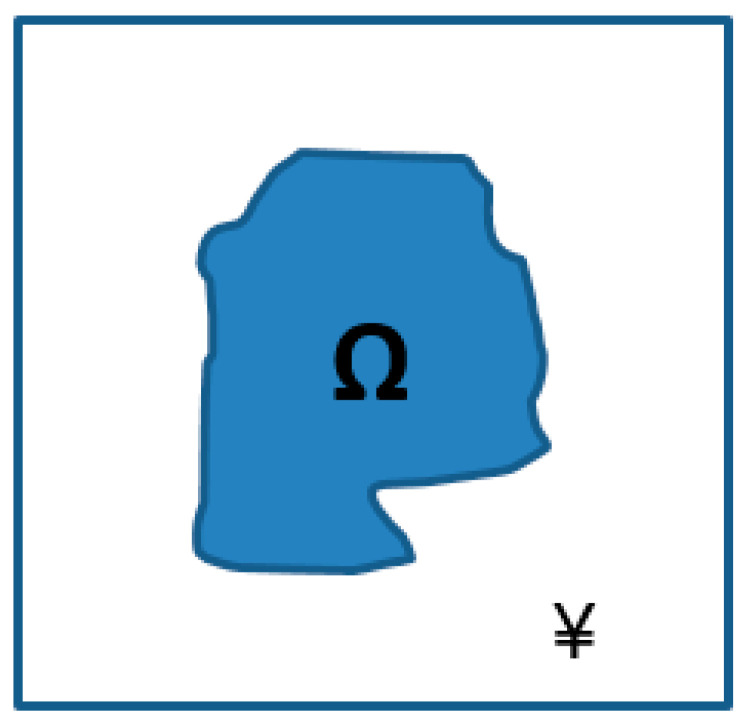
Two elastic subdomain materials (¥ and Ω) within a nanocomposite unit cell. The rigidity coefficient of ¥ and the global rigidity of the unit cell are known.

**Figure 4:**
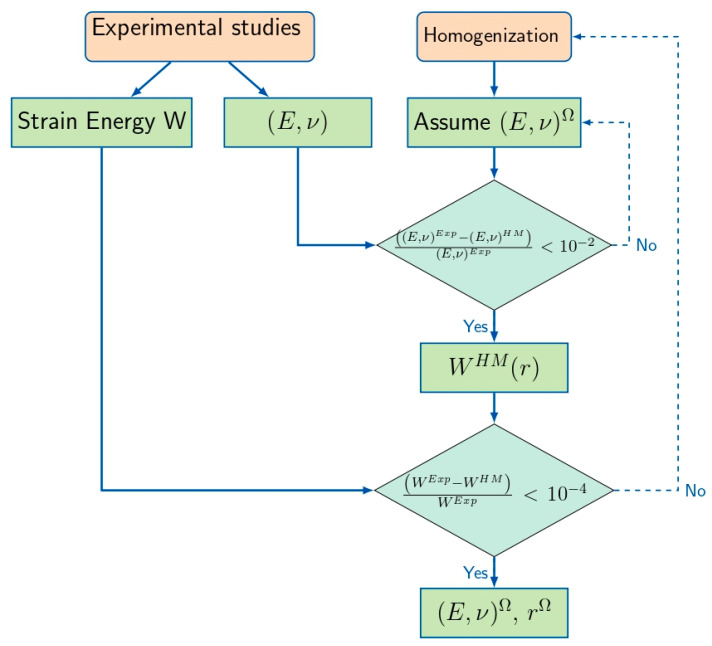
Flowchart describing the experimental–HM bridged multiscale approach, which aims to characterize the local mechanical behavior of a subdomain Ω based on the comparison criteria for elastic properties and strain energies.

We aimed to obtain the mechanical properties for an arbitrary local subdomain Ω (see Figure 3) through a periodic composite matrix using a micro-continuum multi-scale model coupling experimental data and homogenization (HM). Specifically, we used the inverse problem-based approach introduced in [23] by Barakat et al. (2024) and developed for nanocomposite materials. A homogenized model based on a multi-layer unit cell, described in Section 2, that has a periodic microstructure composed of multiple perfectly bonded homogeneous phases was used. Using this framework of homogenization, we calculated the local mechanical properties in the desired subdomain. In Figure 4, we depict a simplified flowchart describing our multiscale bridging algorithm.

In step one, initial estimates of Young’s modulus (E) and Poisson’s ratio (ν) of the subdomain Ω were assigned. These estimates were used in such a way that an initial average stiffness (rigidity) matrix was determined. At the same time, the thickness of the subdomain Ω was also set up. To guarantee faster convergence, these initial values were chosen to be close to the mechanical properties of neighboring phases. Following these values, the homogenization method (HM) was used to determine the effective elastic constants of the whole unit cell. Thus, the generated values were matched to experimental values of global mechanical behavior, as shown here:

(10) $$\frac{\left({\left(E,\nu \right)}^{Exp}\right)-\left({\left(E,\nu \right)}^{HM}\right)}{{\left(E,\nu \right)}^{Exp}}\leq 10^{-2}$$

When the condition mentioned in Equation (10) was not satisfied, the initial values of the components of the rigidity matrix were updated with a difference of 0.2% from the previous values. Thus, we performed a systematic scan of possible values until convergence was reached. Next, we attempted to find the effective thickness of the subdomain Ω. To this end, we calculated the strain energy in the linear elastic regime following homogenization and given by *W*^*HM*^ = 0.5 × **C**^*HM*^:*ϵ*^hom^. This quantity was compared with the experimentally determined strain energy, denoted by *W*^Exp^, which is the area under the linear elastic region of the tensile test stress-strain curve. A discrepancy in this quantity shows that the homogenized model does not properly capture the internal stress distribution in the subdomain, even though it correctly captures the overall stiffness of the entire unit cell. To correct this discrepancy, the radial thickness of the subdomain’s r (being treated as a spherical inclusion) was increased by 0.06. The model was re-meshed and solved again through homogenization.

(11) $$\frac{\left({\left(W\right)}^{Exp}\right)-\left({\left(W\right)}^{HM}\right)}{{\left(W\right)}^{Exp}}\leq 10^{-4}$$

This iterative process is continued until the convergence criterion given by Equation (11) is met. On convergence, the calibrated Young’s modulus, Poisson’s ratio, and subdomain thickness are obtained. These parameters define the mechanical properties of the subdomain Ω. FreeFEM 14.3, an open-source finite element analysis program that excels in resolving partial differential equations in intricate geometries, was used to perform the finite element simulations in this work. For the inverse approach, FreeFEM 14.3 was used in combination with homogenization and inverse modeling techniques to evaluate the effective mechanical properties of the material, such as stiffness and elasticity, based on experimental observations. The computational domain was created using the buildmesh function, which usually uses triangular elements to strike the best possible balance between computational cost and precision (Number of Triangles: 200 Number of Vertices: 120 DOF: 1800). The simulations solve the governing equations of elasticity, with displacement as the primary unknown variable. Effective continuum-scale material properties were extracted by simulating mechanical loads using either macroscopic deformation or a specified gradient of deformation as a boundary condition.

To validate the developed inverse approach, we consider the case of an isotropic metal matrix composite (aluminum AA6061–silicon carbide (SiC)), studied by Wiśniewska et al. [24]. The inclusions are of a spherical shape, with a volume fraction of 40%. All relevant data are provided in Table 1.

The mechanical parameters used in the inverse approach simulation are listed in Table 1. The composite consists of a periodic cubic cell with a length of 1 mm. As outlined in the flowchart, the required experimental results include Young’s modulus and the strain energy (derived from Young’s modulus and Poisson’s ratio). The objective is to determine the properties of the matrix and the corresponding volume fraction of the inclusions (radius). The matrix has an initial Young’s modulus of 20 GPa and a Poisson’s ratio of 0.25, while the inclusions have an initial radius of 0.2 mm, corresponding to a volume fraction of 12.5%. These starting values were selected randomly. The rigidity matrix corresponds to the Voigt representation of the fourth-order tensor for isotropic materials. From this matrix, the values of E (Young’s modulus) and ν (Poisson’s ratio) can be directly obtained using the standard relations:

(12) $$E=\frac{\left(C_{11}-C_{12}\right)\left(C_{11}+2C_{12}\right)}{C_{11}+C_{12}},\;\nu =\frac{C_{12}}{C_{11}+C_{22}}$$

The primary goal is to determine the properties of the matrix inside the composite and to identify the optimal radius of the inclusion. After several iterations of the described method in the flowchart and verification of the conditions for both loops, the code successfully converges, resulting in the following outcomes:

- We reached an inclusion radius of 0.33 mm, corresponding to a volume percentage of 35%;
- The following rigidity matrix was obtained:
  $$\left(\begin{array}{cccccc} 99.7 & 44.8 & 44.8 & 0 & 0 & 0\\ 44.8 & 99.7 & 1555.5 & 0 & 0 & 0\\ 44.8 & 44.8 & 99.7 & 0 & 0 & 0\\ 0 & 0 & 0 & 27.5 & 0 & 0\\ 0 & 0 & 0 & 0 & 27.5 & 0\\ 0 & 0 & 0 & 0 & 0 & 27.5 \end{array}\right)$$

The extracted properties from the above-obtained matrix are Young’s modulus E = 72 GPa and Poisson’s ratio ν = 0.31.

Compared with the matrix data in Table 1, the inverse approach shows very good agreement in computing local properties, with a relative error of 3% for Young’s modulus and 6% for Poisson’s ratio.

In order to improve the applicability of the inverse approach for a broader audience, we are illustrating another example of orthotropic composite material with polymer matrix that demonstrates the robustness and versatility of the inverse homogenization framework. The unidirectional fiber-reinforced composite material used in this study was produced at Izoreel Composite Isolate (Izmir, Turkey) Materials Company. The specimens were made for E-glass fiber and epoxy resin CY225 (with ~60% fiber volume fraction). Tensile tests conducted according to ASTM D3039 measured both longitudinal Young’s modulus (E_1_ ≈ 37.2 GPa), transverse Young’s modulus (E2 ≈ 16.4 GPa) and Poisson’s ratio (ν_12_ ≈ 0.28). All relevant data of the constituents are provide in Table 2 [25].

The goal is to determine the properties of the matrix inside the composite and to identify the optimal radius of the inclusion. After several iterations of the described method in the flowchart and verification of the conditions for both loops, we reached an inclusion radius of 0.45 mm, corresponding to a volume percentage of 63%; The extracted properties are Young’s modulus E = 3.2 GPa and Poisson’s ratio ν = 0.32. Compared with the matrix data in Table 1, the inverse approach shows very good agreement in computing local properties, with a relative error of 6% for Young’s modulus and 6% for Poisson’s ratio.

## 4. Strain Gradient Homogenization Towards Generalized Continuum

To characterize the structure of an effective second-gradient medium, the total microscopic displacement field is broken down additively into a homogeneous component $u^{hom}(y)$, which is affine with the macroscopic strain **E** and its gradient **K**. The homogeneous displacement is the response of an idealized (hypothetical) effective continuum, which would be equivalent to a homogeneous strain-pumped medium. For more details about the development of an effective second-gradient medium, see [26].

This second-gradient continuum is characterized by a bilinear representation of strain energy in terms of the relevant kinematic quantities:

(13) $$\begin{array}{c} W_{M}\left(E,K\right)=\frac{1}{2}E:C^{hom}:E+\frac{1}{2}K∴D^{hom}:K+E:B^{hom}:K=\\ ={\langle w_{\mu }\left(\varepsilon \right)\rangle }_{Y}={\langle \frac{1}{2}\varepsilon \left(y\right):C\left(y\right):\varepsilon \left(y\right)\rangle }_{Y} \end{array}$$

where $D^{\mathrm{hom}}$ is the sixth-order tensor of the strain gradient moduli and $B^{\mathrm{hom}}$ a fifth-order tensor of the coupled first- and second-gradient moduli. This definition results in a mesoscopic strain gradient energy density, which itself provides the constitutive relations at the mesoscopic scale. These connect both the mean Cauchy stress and hyperstress tensors to the macroscopic strain **E** and strain gradient **K**, resulting in the second-gradient constitutive law.

(14) $$\Sigma :=\frac{\partial W_{M}\left(E,K\right)}{\partial E}\equiv C^{hom}:E+B^{hom}∴K;\;S:=\frac{\partial W_{M}\left(E,K\right)}{\partial E}\equiv B^{\mathrm{hom},\;T,}:E+D^{hom}∴K$$

**S** is the hyper stress. Note that the coupling terms between the first and second gradients present in (14) vanish for centrosymmetric media. An extended minimization principle over all periodic fluctuations holds, so one can write:

(15) $$W_{M}\left(E,K\right)=\underset{\tilde{u}\in H_{per}^{1}(Y)}{Min}\left\{\int_{Y}\frac{1}{2}C\left(y\right):\left(E+K:y+\tilde{u}(y)⊗\nabla_{y}\right):\left(E+K:y+\tilde{u}(y)⊗\nabla_{y}\right)dV_{y}\right\}$$

Using the Hill lemma again, the average Cauchy stress tensor defined in the second set of relations (14) writes

(16) $$\Sigma_{ij}=\frac{\partial W_{M}}{\partial E_{ij}}=\frac{\partial }{\partial E_{ij}}{\langle w_{\mu }\left(\varepsilon \right)\rangle }_{Y}={\langle \frac{\partial w_{\mu }\left(\varepsilon \right)}{\partial E_{ij}}\rangle }_{Y}={\langle \frac{\partial w_{\mu }\left(\varepsilon \right)}{\partial \varepsilon_{pq}}\frac{\partial \varepsilon_{pq}}{\partial E_{ij}}\rangle }_{Y}={\langle \sigma_{pq}\frac{\partial \varepsilon_{pq}}{\partial E_{ij}}\rangle }_{Y}$$

Similarly, the average macroscopic hyperstress defined in the second set of Equation (14) writes using the Hill lemma as

(17) $$S_{ijk}=\frac{\partial W_{M}}{\partial K_{ijk}}=\frac{\partial }{\partial K_{ijk}}{\langle w_{\mu }\left(\varepsilon \right)\rangle }_{Y}={\langle \frac{\partial w_{\mu }\left(\varepsilon \right)}{\partial K_{ijk}}\rangle }_{Y}={\langle \frac{\partial w_{\mu }\left(\varepsilon \right)}{\partial \varepsilon_{pq}}\frac{\partial \varepsilon_{pq}}{\partial K_{ijk}}\rangle }_{Y}={\langle \sigma_{pq}\frac{\partial \varepsilon_{pq}}{\partial K_{ijk}}\rangle }_{Y}$$

The detailed computations leading to the expression of the microscopic homogeneous displacement versus the macroscopic strain and strain gradient tensors are detailed in [26].

These computations deliver the expression of the homogeneous part of the microscopic displacement versus the macroscopic strain and strain gradient tensors (for more details, please see Appendix A):

(18) $$u^{hom}=\left|\begin{array}{c} u_{1}^{hom}=Ky_{2}+E_{11}y_{1}+E_{12}y_{2}+K_{111}\frac{y_{1}^{2}}{2}+K_{112}y_{1}y_{2}+\left(K_{122}+K_{212}-K_{221}\right)\frac{y_{2}^{2}}{2}\\ u_{2}^{hom}=-Ky_{1}+E_{21}y_{1}+E_{22}y_{2}+\left(K_{121}+K_{211}-K_{112}\right)\frac{y_{1}^{2}}{2}+K_{221}y_{1}y_{2}+K_{222}\frac{y_{2}^{2}}{2} \end{array}\right.$$

The optimal boundary for the BVP associated with the real displacement field (in the absence of body forces) is a necessary condition that must be satisfied according to the stationarity conditions of the functional on the right-hand side of (15):

(19) $$\left|\begin{array}{c} -div_{y}\left\{C\left(y\right):\left(E+K:y+\tilde{u}(y)⊗\nabla_{y}\right)\right\}=0\\ \tilde{u}(y)\;Y-periodic \end{array}\right.$$

Since second-gradient media are sensitive to the gradient of the deformation field, the choice of spatial reference frame becomes highly significant. Consequently, the selection of the global reference point has a direct impact on the computed effective properties. In the original work in [26], the numerical implementation assumed the corner point of the cubic unit cell as the origin of the reference frame. This choice led to a high percentage of fluctuation energies, potentially affecting the accuracy of the homogenized second-gradient properties. In this work, we extend their theory by considering the center of mass of the unit cell as the reference coordinate for the numerical implementation. The macroscopic energy density leads to the following strain gradient constitutive law at the macroscopic level for centrosymmetric media, relating the average Cauchy stress and hyperstress to the strain and strain gradient tensors expressed in index and tensor form (notice that the coupled terms vanish for centrosymmetric media):

(20) $$\left(\begin{array}{c} \Sigma_{11}\\ \Sigma_{22}\\ {\sqrt{2}\Sigma }_{12} \end{array}\right)=\left(\begin{array}{ccc} c_{1111}^{hom} & c_{1122}^{hom} & \sqrt{2}c_{1112}^{hom}\\ c_{2211}^{hom} & c_{2222}^{hom} & {\sqrt{2}c}_{2212}^{hom}\\ {\sqrt{2}c}_{1211}^{hom} & {\sqrt{2}c}_{1222}^{hom} & c_{1212}^{hom} \end{array}\right)\left(\begin{array}{c} E_{11}\\ E_{22}\\ {\sqrt{2}E}_{12} \end{array}\right)\;\left(\begin{array}{c} S_{111}\\ S_{221}\\ \sqrt{2}S_{122}\\ S_{222}\\ S_{112}\\ \sqrt{2}S_{121} \end{array}\right)\;=\left(\begin{array}{cccccc} & D_{111221}^{hom} & \sqrt{2}D_{111122}^{hom} & D_{111222}^{hom} & D_{111112}^{hom} & \sqrt{2}D_{111121}^{hom}\\ D_{221111}^{hom} & D_{221221}^{hom} & \sqrt{2}D_{221122}^{hom} & D_{221222}^{hom} & D_{221112}^{hom} & \sqrt{2}D_{221121}^{hom}\\ \sqrt{2}D_{122111}^{hom} & \sqrt{2}D_{122221}^{hom} & 2D_{122122}^{hom} & \sqrt{2}D_{122222}^{hom} & \sqrt{2}D_{122112}^{hom} & 2D_{122121}^{hom}\\ D_{222111}^{hom} & D_{222221}^{hom} & \sqrt{2}D_{222122}^{hom} & D_{222222}^{hom} & D_{222112}^{hom} & \sqrt{2}D_{222121}^{hom}\\ D_{112111}^{hom} & D_{112221}^{hom} & \sqrt{2}D_{112122}^{hom} & D_{112122}^{hom} & D_{112112}^{hom} & \sqrt{2}D_{112121}^{hom}\\ \sqrt{2}D_{121111}^{hom} & \sqrt{2}D_{121221}^{hom} & 2D_{121112}^{hom} & \sqrt{2}D_{121122}^{hom} & \sqrt{2}D_{121112}^{hom} & 2D_{121121}^{hom} \end{array}\right)\left(\begin{array}{c} K_{111}\\ K_{221}\\ \sqrt{2}K_{122}\\ K_{222}\\ K_{112}\\ \sqrt{2}K_{121} \end{array}\right)$$

We propose a numerical algorithm consistent with the proposed theoretical framework exposed in previous section to determine the effective rigidity tensors of the homogenized second-gradient constitutive law. We introduce the macroscopic deformation and gradient of deformation, tensors E, K, such that the microscopic deformation tensor can accordingly be decomposed into its homogeneous and fluctuating part:

(21) $$\begin{array}{c} \varepsilon \left(y\right)=\varepsilon^{hom}\left(y\right)+\tilde{\varepsilon }\left(y\right),\\ \varepsilon^{hom}(y):=u^{hom}(y){⊗}^{S}\nabla_{y}=E+K.\;y,\;\forall y\in Y \end{array}$$

Applying the macroscopic deformation E alone as a kinematic boundary condition over the RVE leads to the first effective rigidity matrix $C^{\mathrm{hom}}$, while applying the gradient of deformation K alone as a kinematic boundary condition over the RVE entails the evaluation of coupling matrix $B^{\mathrm{hom}}$ and the second rigidity matric. Using the expression in Equation (19), we obtain the second-gradient rigidity matrix $D^{\mathrm{hom}}$ completing the set of effective rigidity tensors.

The macroscopic deformation E and the macroscopic gradient of deformation K being the kinematic controls applied to the unit cell Y, we search for the total displacement u of the unit cell, relying on a decoupling between the pure first gradient and a coupling between the first and second-gradient problems. The total microscopic displacement u satisfies the following set of governing equations:

(22) $$\left|\begin{array}{c} div_{y}\sigma (y)=0\;in\;Y\\ \sigma (y)=C(y):\varepsilon (y)\;in\;Y\\ \varepsilon (y)=E+K\cdot y+\tilde{\varepsilon }(y)\\ \tilde{\varepsilon }(y):=\tilde{u}(y){⊗}^{S}\nabla_{y} \end{array}\right.$$

with $\tilde{\varepsilon }(y)$ therein the fluctuating part of the microscopic deformation. The Cauchy effective rigidity tensor is evaluated from Equation (16) by selecting K=0 and the tensor of coupling moduli is obtained by selecting E=0 in Equation (16) and evaluating the average of the microscopic Cauchy stress tensor, according to Equation (17) which is expressed versus E and K.

We find as the solution of Equation (14) the fluctuating displacement field $\tilde{u}$ associated to the elastic matrix C(y), as the solution of the following formal homogenized problems written in variational format, considering the decomposition of the total displacement, $u=E+K\cdot y+\tilde{u}$:

(23) $$\forall v\in H_{1}^{\#}\left(Y\right),\int_{Y}C(y):\varepsilon (u):\varepsilon (v)dV_{y}=0\;$$

The problem resumes to find the periodic fluctuating displacement $\tilde{u}$ satisfying the variational equation associated to the elastic part of the previous BVP successively written in Equation (19) and leading to the first effective rigidity matrix $C^{\mathrm{hom}}$ when applying the deformation E, and to the coupling matrix $B^{\mathrm{hom}}$ when applying the gradient of deformation via tensor **K**, such that the following variational equation is satisfied as the consequence of Equation (15):

(24) $$\int_{Y}C(y):\varepsilon (\tilde{u}):\varepsilon (v)dy=-\int_{Y}C(y):\left(E+K.y\right):\varepsilon (v)dV_{y},\;\forall v\in H_{1}^{\#}\left(Y\right)$$

The determination of the periodic function $\tilde{u}\in Y$ allows calculating the homogenized elastic tensors C^hom^ and B^hom^ associated to the microscopic tensors C(y), which receive the following expressions:

(25) $$\begin{array}{c} K=0\to \Sigma :=C^{hom}:E=\frac{1}{\left|Y\right|}\int C(y):\varepsilon (u)dV_{y}\\ E=0\to \Sigma =B^{hom}∴K=\frac{1}{\left|Y\right|}\int C(y):\varepsilon (u)dV_{y} \end{array}$$

Using Equation (15) involving the second-gradient displacement localizators, we finally evaluate the second-gradient rigidity matrix $D^{\mathrm{hom}}$.

In order to investigate the accuracy when modeling the composite with a second-gradient continuum, the relative contribution of the fluctuation energy over the total microscopic energy for the successive components of the Cauchy strain and strain gradient kinematic measures are evaluated. Table 3 shows the macroscopic, microscopic, and fluctuation energies for a unit cell with a fiber radius of R=0.25 mm. These energetic terms can be successively written as follows:

$$W_{Macro}=\frac{1}{2}\left(\Sigma :E+S∴K\right);\;W_{micro}=\frac{1}{2}\left(\sigma :\varepsilon \right);\;W_{fluctuation}=\frac{1}{2}(\sigma :\varepsilon (\tilde{u}))$$

As shown in the above table, the values of the microscopic, macroscopic, and fluctuation energies satisfy the relation $W_{micro}=W_{Macro}+W_{fluctuation}$, where the Hill–Mandel lemma is satisfied. The low value of the fluctuation energy in Table 3 implies that microstructural heterogeneities have lower influence on the global mechanical response of the composite, which can indicate several key factors. First, the proposed second-order homogenization approach captures the microstructural behavior well, where the strain field remains close to its effective value and local fluctuations are suppressed. Additionally, in the context of strain gradient theory, low fluctuation energy may signify the dominance of higher-order stiffness contributions, where microstructural length-scale effects play a crucial role in stabilizing the system. Lower fluctuation energy means that the microstructure causes less deviation from the assumed homogeneous deformation. This indicates that the microstructural heterogeneities (e.g., inclusions and pores) have a weaker influence on the global mechanical response. Furthermore, it can reflect weak local heterogeneities, as a small contrast between phases or microstructural elements, such as inclusions or voids, results in less pronounced strain localization. Lastly, it may indicate efficient energy redistribution, where mechanical energy is well distributed without significant localizations, suggesting a well-dispersed microstructure or optimal coupling between different length scales.

**Figure 5:**
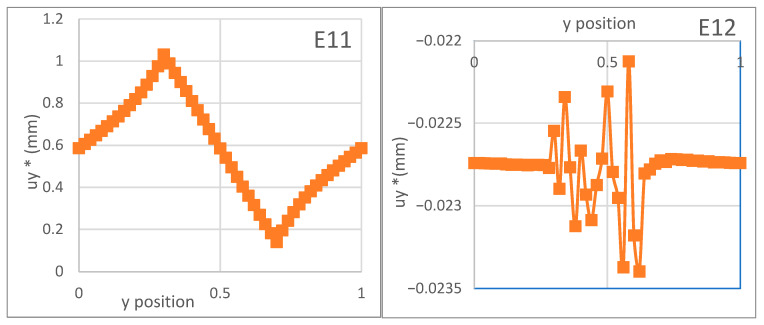
Distribution of fluctuating displacement $\tilde{u}$ (in mm) on the vertical axis due to the macroscopic strains $E_{11}=1$ and $E_{12}=1$ along a vertical line through the centers of the inclusions.

Figure 5 elaborates on the distribution of fluctuating displacement components due to externally applied macroscopic strains E_11_ = 1 and E_12_ = 1, calculated along a vertical axis bisecting the centers of the inclusions in the irreducible unit cells. Under both macroscopically applied loads, the displacement fluctuations at the microscopic level are extremely pronounced relative to the inclusions. However, the fluctuations associated with shear deformation occur significantly less frequently and are of a lower magnitude. The displacement component ${u^{*}}_{y,1}$ is patterned after the unit cell structure through corresponding internal unit cells. Thus, regardless of whether the calculations are carried out for a singular unit, the effective Cauchy properties continue to remain consistent.

**Figure 6:**
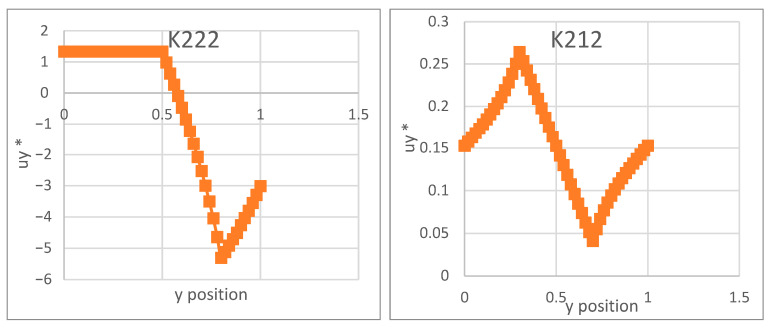
Distribution of displacement fluctuation ${\tilde{u}}_{1}$ (mm) due to the macroscopic gradient of deformation *K*_111_ = 1 and K_212_ = 1 along a vertical line that passes through the centers of the inclusions.

The higher-order problem is then considered, where a macroscopic deformation gradient of K_111_ and K_212_ is applied independently to the unit cell, analogous to the Cauchy-type localization analysis, as discussed here. As shown in Figure 6, the amplitude of oscillatory displacement experiences a broad range of variations inside the inclusion, where the high contrast in the material properties demonstrates the potential impact of the y microstructure. The amplitude of the displacement fluctuations, moreover, is reduced by factors of 2 and 3 when the analysis is extended to windows containing four and nine irreducible unit cells, respectively. As a consequence, the components of the second-order rigidity matrix vary linearly with the number of unit cell repetitions N when computations are conducted over many repetitive periodic cells. This highlights that the effective second-gradient moduli are independent of the number of unit cells and can, therefore, be determined using the irreducible unit cell, similar to the classical Cauchy-type elastic moduli.

**Figure 7:**
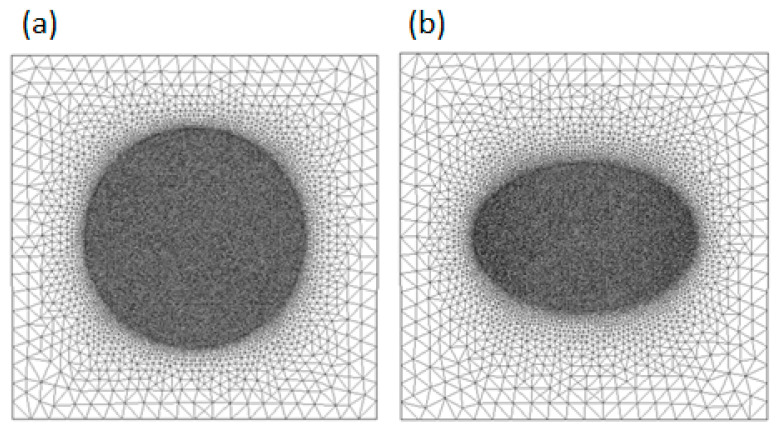
The two studied microstructures, with (**a**) circular and (**b**) elliptical model fiber shapes within the square matrix domain.

In this section, we compare two different microstructures associated with different fiber geometries: the first one has a circular shape, and the second is an elliptical fiber shape, with both surrounded by a square matrix (Figure 7). For the two microstructures, the outer shape designates the matrix and the inner shape represents the inclusion. The mechanical properties of both the inclusions and the matrix are provided in Table 4.

**Figure 8:**
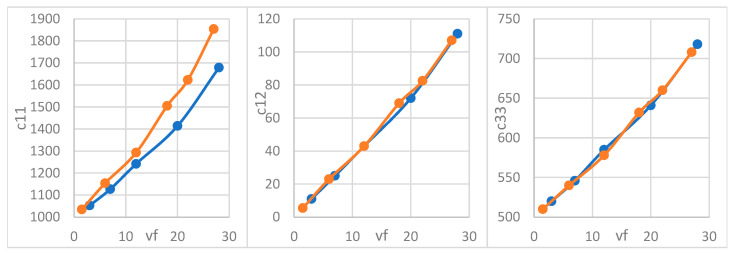
Rigidity coefficient as a function of the volume percentage of fiber. The orange line represents the elliptical shape, and the blue one represents the circular shape (units GPa).

**Figure 9:**
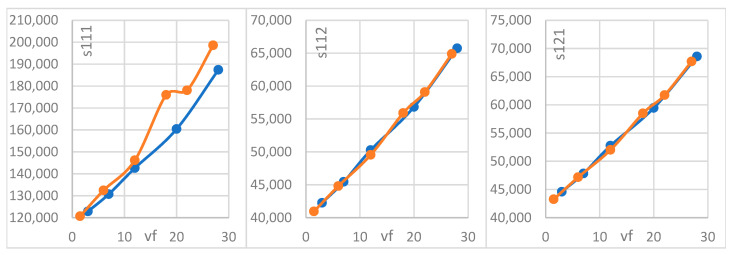
Second-gradient coefficient as a function of the volume percentage of fiber. The orange line represents the elliptical shape, and the blue one represents the circular shape (units GPa.mm^2^).

In Figure 8 and Figure 9, we depict the dependence of the homogenized coefficients for the rigidity coefficients ${\bar{C}}_{11},\;{\bar{C}}_{12}$, and ${\bar{C}}_{33}$, as well as for the second-gradient coefficients $S_{111},\;S_{112},\;\mathrm{and}\;S_{121}$ as functions of the volumetric content $\nu_{f}$, with the two phases in Figure 6 (circular and elliptical fiber shapes). Increasing the volumetric percentage of fiber leads to an increase in the first-, second-, and coupled-rigidity coefficients, as shown in Figure 7 and Figure 8.

Figure 8 and Figure 9 indicates a major influence of fiber shape on the rigidity coefficient. The relative difference reaches a maximum value of 9%. This behavior is due to the fillet for the unit cell showing more second-gradient effects. For the limiting values of the layer contents $\nu_{f}$ ($\nu_{f}\to 0,\;\nu_{f}\to 1$), the method asymptotically converges to the properties of individual layers for the first-gradient rigidity coefficients, validating the obtained results.

## 5. Wave Propagation Analysis in the Second-Gradient Medium

For an effective homogenized 2D second-gradient medium, the dynamical equations of motion can be written in component form along the x and y directions of a Cartesian coordinate system as the two following differential equations [27]:

(26) $$\left(\frac{\partial \sigma_{11}}{\partial x_{1}}+\frac{\partial \sigma_{12}}{\partial x_{2}}\right)-\frac{\partial^{2}S_{111}}{\partial x_{1}\partial x_{1}}-\frac{\partial^{2}S_{112}}{\partial x_{1}\partial x_{2}}-\frac{\partial^{2}S_{121}}{\partial x_{2}\partial x_{1}}-\frac{\partial^{2}S_{122}}{\partial x_{2}\partial x_{2}}=\rho^{*}\ddot{u}$$

(27) $$\left(\frac{\partial \sigma_{21}}{\partial x_{1}}+\frac{\partial \sigma_{22}}{\partial x_{2}}\right)-\frac{\partial^{2}S_{211}}{\partial x_{1}\partial x_{1}}-\frac{\partial^{2}S_{212}}{\partial x_{1}\partial x_{2}}-\frac{\partial^{2}S_{221}}{\partial x_{2}\partial x_{1}}-\frac{\partial^{2}S_{222}}{\partial x_{2}\partial x_{2}}=\rho^{*}\ddot{v}$$

where $\ddot{u}$ and $\ddot{v}$ are the horizontal and vertical components of the acceleration vector. The effective density ρ is given by the average density between the inclusion and the matrix obtained through the mixture rule. In order to obtain the displacement formula for the equations of motion, the compatibility equations involving the macro-strain components are written in function of the displacements as follows:

$$\epsilon_{11}=u_{,x},\;\epsilon_{22}=v_{,y},\;\epsilon_{12}=u_{,y},\epsilon_{21}=v_{,x}$$

The gradient of deformation is given by its six independent components, as follows:

$$K_{111}=u_{,xx},\;K_{222}=v_{,yy},\;K_{121}=u_{,yx},K_{212}=v_{,yx},K_{122}=u_{,yy},K_{211}=v_{,xx}$$

The gradient of deformation is used in the constitutive equation, so Equations (26) and (27) are expressed in terms of displacement and first- and second-rigidity matrices. For a harmonic wave propagating along an axis in an infinite planar second-gradient medium, the generalized displacement field with components u and v at a point r is assumed to be in the following form:

(28) $$u=\hat{u}\;e^{\left(iwt-i\;k\;.r\right)}$$

(29) $$v=\hat{v}\;e^{\left(iwt-i\;k\;.r\right)}$$

where $\hat{u}\;\mathrm{and}\;\hat{v}$ are wave amplitudes, $k=\left(k_{1},k_{2}\right)$ is the wave vector (each component is a complex number), **r** is the position, and w is a frequency function (function of the wave vector) that permits wave attenuation in space. Substituting Equations (26) and (27) in the equations of motion in (21) and (22) results in the following algebraic equation, which represents the wave motion equation:

(30) $$\left[D\left(k_{1},k_{2},w\right)\right]\left(\begin{array}{c} \;\hat{u}\\ \hat{v} \end{array}\right)=0$$

The wave vector **k** is a complex number: its real part represents attenuation in the x–y plane, and its imaginary part is the phase constants. For a plane wave without attenuation in the x–y plane, the propagation constants along the x and y directions are $k_{1}=i\varepsilon_{1}=\left|k\right|cos\left(\theta \right)$ and $k_{2}=i\varepsilon_{2}=\left|k\right|sin(\theta )$. All triads of $k_{1},k_{2},\mathrm{and}\;w$ obtained by solving the eigenvalues in (25) represent plane waves propagating at frequency w and in the longitudinal and shear modes.

### 5.1. Comparison Between Cauchy-Type Material and Effective Second-Gradient Medium

A wave propagation analysis was performed for a unit cell with the data in Table 3 and with a fiber radius of R=0.35 mm (spherical inclusion). The constitutive law was first formulated based on the equation in Section 4 to derive the Cauchy rigidity matrix C and the second-gradient rigidity matrix D. Then, the wave motion Equation (30) was applied to determine the two propagation modes.

**Figure 10:**
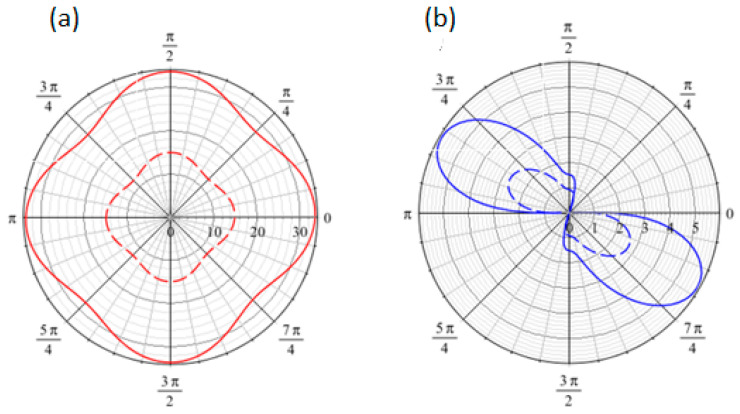
Angular dispersion relation for (**a**) longitudinal and (**b**) shear waves for k = 1 (dashed) and k = 1.5 (continuous).

The irregular shape of the angular dispersion curves in the two propagation modes (longitudinal and shear) shown in Figure 10 highlights the anisotropic behavior of the 2D composite structure with a circular inclusion. An increase in the wavenumber *k* results in increases in dispersion relation in both modes, as illustrated in Figure 10, which reflects the dispersive behavior characteristic of the second-order gradient continuum and the compatibility with the experimental finding. Note that a similar behavior was observed regardless of the angle, indicating that this behavior is consistent across different propagation directions.

**Figure 11:**
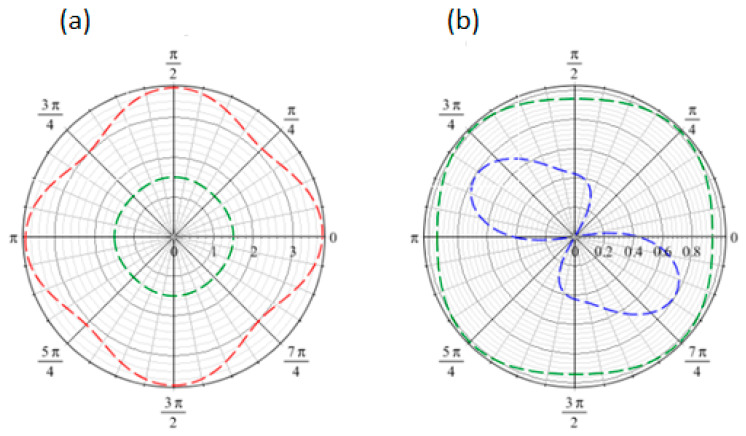
Angular dispersion relation for (**a**) longitudinal and (**b**) shear waves for second-gradient (red) and Cauchy (green, blue) media.

Figure 11 compares the dispersion relations for longitudinal and shear modes in Cauchy and second-gradient media. An inspection of Figure 11 shows significant variations between the two media. The Cauchy medium, defined by circular dispersion laws for both modes, does not capture anisotropic behavior, implying isotropic behavior and contradicting the experimental results. In contrast, the second-gradient medium properly depicts anisotropy via irregular dispersion relations, revealing the wave propagation’s directional dependence inside the medium. Furthermore, the longitudinal mode in the second-gradient medium has greater values than the Cauchy medium, indicating enhanced stiffness caused by microstructural effects and emphasizing the effect of the second-gradient rigidity matrix D. In contrast, the shear mode exhibits lower values in the second-gradient medium, indicating the ability of higher-order gradients to account for localized compliance or deformation mechanisms where the classical Cauchy framework fails.

### 5.2. Effect of Inclusion Shape and Volume Percentage

In this section, we investigate the influence of the volume percentage and shape of the inclusion on the longitudinal and shear modes. Two inclusion shapes, spherical and elliptical (as shown in Figure 6), are considered, with the volume percentage varying from 0% to 30%. The wavenumber is set to 1.5 m^−1^, with an angular direction of 45°.

**Figure 12:**
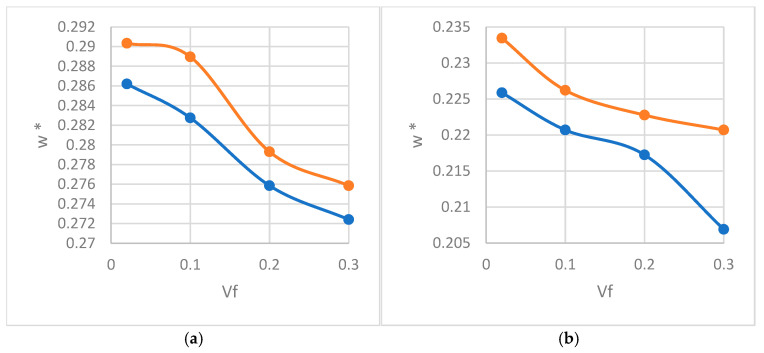
Normalized dispersion relation $w^{*}=w/\sqrt{E_{m}/\rho_{m}}$ for (**a**) longitudinal and (**b**) shear waves for circular (blue) and elliptical (orange) inclusions.

As the volume percentage decreases in Figure 12, both modes of propagation (longitudinal and shear) decrease for both inclusion shapes (circular and elliptical) due to the increase in both the first- and second-gradient rigidity matrices. Elliptic inclusions present higher modes of propagation compared with circular inclusions due to their geometrical anisotropy, which creates directional differences in stiffness along the major and minor axes and stress concentrations around the inclusions. This anisotropy results in a more complex stress and strain distribution, enhancing phase velocity and increasing the modes of propagation. Additionally, the elongated shape of the elliptical inclusions leads to more complex interfacial interactions with the matrix, further contributing to higher-frequency modes. In contrast, circular inclusions exhibit more isotropic behavior, resulting in lower propagation modes.

### 5.3. Effect of Contrast Between Properties

In this section, we shed light on the effect of contrast between the properties of inclusions and matrices in both modes of propagation ($E_{inc}/E_{mat}$). For this reason, we work on a circular inclusion with a volume percentage of 30% and the wavenumber is set to 1.5 m^−1^, with an angular direction of 45°.

**Figure 13:**
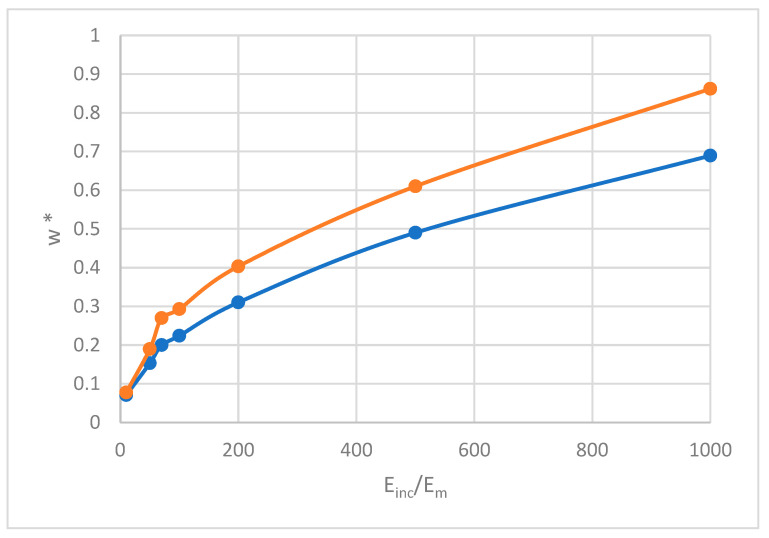
Normalized dispersion relation $w^{*}=w/\sqrt{E_{m}/\rho_{m}}$ for longitudinal (orange) and shear (blue) waves as a function of the contrast between properties $E_{inc}/E_{mat}$.

As observing in Figure 13, increasing the property contrast between the inclusion and the matrix significantly enhances longitudinal and shear propagation modes. Notably, this increase is non-monotonic. The increase in both propagation modes is due to a stronger stiffness mismatch, which alters stress wave interactions, and enhances wave scattering and reflection at the inclusion–matrix interphase. A stiffer inclusion increases the first and second effective rigidity matrices, increasing the wave phase velocity of waves, while microstructural interactions in second-gradient materials further amplify this effect. However, the increase is non-monotonic, as excessive contrast can lead to localized stress concentrations or mode coupling effects that may either enhance or reduce propagation depending on the wave interactions and microstructure effect.

## 6. Conclusions and Perspectives

The aim of this work was to develop an effective strain gradient constitutive law to replace the heterogeneous composite material. The homogenization approach was based on the Hill lemma in combination with a variational approach. In addition, the microscopic deformation was decomposed into a periodic fluctuation that arises from the local heterogeneities and a homogeneous part, which reproduce the global deformation applied to the material.

We first presented the classical homogenization theory for Cauchy-type media. Then, we developed an inverse approach to determine local properties for any subdomains in composite materials given known global properties. We tested the validity of this approach using experimental data for a metal matrix composite (aluminum AA6061–silicon carbide (SiC)). The approach showed good agreement when predicting the properties of the aluminum metal matrix and the volume percentage of the inclusion. This approach can be incorporated as a “test-before-invest” methodology as a critical early-stage evaluation tool to optimize composite materials for tailored applications. Table 5 shows the good agreement between the proposed inverse approaches the experimental results for the Young’s modulus E and the Poisson’s ratio ν.

Then, we computed the percentage of fluctuation energy from the macroscopic strain energy for different global deformations in an effective second-gradient medium. The values of the fluctuating, microscopic, and macroscopic energies satisfied the Hill lemma, with smaller deviations in the microscopic energy from the macroscopic one (i.e., the percentage of fluctuating energy was small compared with that of the macroscopic energy).

From the strain gradient constitutive law, we further analyzed the wave propagation in order to study the effect of the strain gradient. After substituting the constitutive law into the dynamical equilibrium equation, we used the harmonic planar wave as a solution, and we obtained the dispersion longitudinal and shear waves.

Increasing the wavenumber, the dispersion relations for both modes increased in a second-gradient medium, where the results were compatible with the experimental results (dispersive phenomena). From the angular shape of the dispersion relation, we examined the isotopic behavior of the shear mode while the longitudinal mode showed anisotropic behavior. We examined the effects of inclusion shape (circular vs. elliptic) and volume percentage on wave propagation, revealing that elliptical inclusions lead to a slight increase in both propagation modes due to their anisotropic geometry, which enhances wave interactions and stiffness distribution within the composite. Additionally, the variation in volume percentage significantly affects the dispersion of waves within the composite, with stiffer inclusion content generally increasing rigidity and altering wave dispersion characteristics. Finally, we investigated the impact of property contrast between the inclusion and matrix, demonstrating its critical role in wave propagation behavior. A higher contrast leads to stronger wave scattering and reflection at the interface, resulting in non-monotonic variations in natural frequency and dispersion properties. This study highlighted the complex interplay between microstructural features and wave dynamics in composite materials.

Future work shall analyze nonlinear wave propagation within the composite material due to geometric nonlinearities arising from to the softening of the matrix phase. Additionally, the development of a homogenization technique that takes into account higher-order inertia terms is of high importance in analyzing higher-frequency modes. Artificial intelligence and machine learning techniques could be integrated to accelerate the inverse homogenization process and optimize microstructural design. These data-driven approaches offer promising tools for predicting local properties from global responses with reduced computational cost.

## Figures and Tables

**Table 1:** Mechanical properties of the inclusion (SiC), matrix (AA 6061), and composite.

Material	AA 6061	SiC	Composite AA6061-SiC
Young’s modulus E	70 Gpa	450 Gpa	140 Gpa
Poisson ratio ν	0.33	0.17	0.25

**Table 2:** Mechanical properties of the E-glass/epoxy composite material.

Material	Epoxy	E-Glass
Young’s modulus E	3 Gpa	72 Gpa
Poisson ratio ν	0.34	0.25

**Table 3:** Comparison between macroscopic and microscopic energies, and contribution of the fluctuation energy.

Applied Component	Macroscopic Energy (N)	Total Microscopic Energy (N)	Fluctuation Energy (N)	Contribution of the Fluctuation (%)
$E_{11}$	621.3	627.18	5.83	0.9
$E_{22}$	619.5	627.04	7.46	1.1
$E_{12}$	292.7	294.9	2.196	0.7
$E_{21}$	292.7	294.9	2.196	0.7
$K_{111}$	71,311.18	71,901.07	589.89	0.8
$K_{212}$	67.9	68.59	0.6	0.9
$K_{221}$	67.9	68.59	0.6	0.9
$K_{112}$	67.9	68.59	0.59	0.86
$K_{211}$	74,329.1	74,848.8	519.7	0.7
$K_{222}$	71,407.73	71,887.5	479.76	0.6

**Table 4:** Mechanical parameters and the density of the fibers and matrix.

	Fibers	Matrix
Elastic modulus	69 GPa	1 GPa
Poisson’s coefficient	0.2	0.33
Density	3190 kg/m^3^	1180 kg/m^3^

**Table 5:** Comparison of mechanical parameters between simulation and experimental.

	Young’s Modulus (E)	Poisson’s Ratio (ν)
Inverse approach	72 GPa	0.31
Experimental	70 GPa	0.33

## Data Availability

The original contributions presented in this study are included in the article. Further inquiries can be directed to the corresponding author.

## Appendix A. Computation of the Homogeneous Microscopic Displacement in the Second-Gradient Medium Imposed over the RVE

For a heterogeneous medium with two phases α,β, for any quantity $\Psi_{\alpha }$ associated with the α-phase (a volume density), the spatial average of the spatial microscopic gradient of $\Psi_{\alpha }$ is given by the macroscopic gradient of the volume average ${\langle \Psi_{\alpha }⊗\nabla_{y}\rangle }_{V_{a}}$ to which one adds a boundary integral evaluated over the interface $I_{\alpha ,\beta }$ common to both phases:

(A1) $${\langle \psi_{\alpha }⊗\nabla_{y}\rangle }_{V_{\alpha }}={\langle \psi_{\alpha }\rangle }_{V_{\alpha }}⊗\nabla_{x}+\frac{1}{V}\int_{I_{\alpha \beta }}n_{\alpha \beta }.\psi_{\alpha }dS$$

The averaging volume V is a control volume (not necessarily periodic) enclosing both phases α,β and vector $n_{\alpha \beta }$ is the unit normal to the α-phase pointing in the direction of the β-phase. Summation of relation (12) written over both phases and using the continuity of $\Psi_{\alpha }$ and the relation $n_{\alpha \beta }+n_{\beta \alpha }$ leads to the final version of Whitaker’s theorem:

(A2) $$\sum_{\delta =1,2}{\langle \psi ⊗\nabla_{y}\rangle }_{V_{\delta }}=\sum_{\delta =1,2}{\langle \psi_{\alpha }\rangle }_{V_{\delta }}⊗\nabla_{x}$$

Relations (A1) and (A2) entail the following system of three algebraic equations in the present planar situation, using the symmetry of the microscopic stress and strain tensors as well as the symmetry of the macroscopic strain:

(A3) $$\left\{\begin{array}{l} \int_{Y}(\sigma_{11}\left(1-\frac{\partial \varepsilon_{11}}{\partial E_{11}}\right)-2\sigma_{12}\frac{\partial \varepsilon_{12}}{\partial E_{11}}-\sigma_{22}\frac{\partial \varepsilon_{22}}{\partial E_{11}})dV_{y}=0,\\ \int_{Y}(-\sigma_{11}\frac{\partial \varepsilon_{11}}{\partial E_{12}}-2\sigma_{12}\frac{\partial \varepsilon_{12}}{\partial E_{12}}+\sigma_{12}-\sigma_{22}\frac{\partial \varepsilon_{22}}{\partial E_{12}})dV_{y}=0,\\ \int_{Y}(-\sigma_{11}\frac{\partial \varepsilon_{11}}{\partial E_{22}}-2\sigma_{12}\frac{\partial \varepsilon_{12}}{\partial E_{22}}-\sigma_{22}\left(1-\frac{\partial \varepsilon_{22}}{\partial E_{22}}\right))dV_{y}=0 \end{array}\right.$$

Relation (A2) entails the following system of eight algebraic equations in the present planar situation:

(A4) $$\left\{\begin{array}{l} \left(1,1,1\right):\int_{Y}(\sigma_{11}y_{1}-\sigma_{11}\frac{\partial \varepsilon_{11}}{\partial K_{111}}-2\sigma_{12}\frac{\partial \varepsilon_{12}}{\partial K_{111}}-\sigma_{22}\frac{\partial \varepsilon_{22}}{\partial K_{111}})dV_{y}=0,\\ \left(1,1,2\right):\int_{Y}(\sigma_{11}y_{2}-\sigma_{11}\frac{\partial \varepsilon_{11}}{\partial K_{112}}-2\sigma_{12}\frac{\partial \varepsilon_{12}}{\partial K_{112}}-\sigma_{22}\frac{\partial \varepsilon_{22}}{\partial K_{112}})dV_{y}=0,\\ \left(1,2,1\right):\int_{Y}(\sigma_{12}y_{1}-\sigma_{11}\frac{\partial \varepsilon_{11}}{\partial K_{121}}-2\sigma_{12}\frac{\partial \varepsilon_{12}}{\partial K_{121}}-\sigma_{22}\frac{\partial \varepsilon_{22}}{\partial K_{121}})dV_{y}=0,\\ \left(1,2,2\right):\int_{Y}(\sigma_{12}y_{2}-\sigma_{11}\frac{\partial \varepsilon_{11}}{\partial K_{122}}-2\sigma_{12}\frac{\partial \varepsilon_{12}}{\partial K_{122}}-\sigma_{22}\frac{\partial \varepsilon_{22}}{\partial K_{122}})dV_{y}=0,\\ \left(2,1,1\right):\int_{Y}(\sigma_{12}y_{1}-\sigma_{11}\frac{\partial \varepsilon_{11}}{\partial K_{211}}-2\sigma_{12}\frac{\partial \varepsilon_{12}}{\partial K_{211}}-\sigma_{22}\frac{\partial \varepsilon_{22}}{\partial K_{211}})dV_{y}=0,\\ \left(2,1,2\right):\int_{Y}(\sigma_{12}y_{2}-\sigma_{11}\frac{\partial \varepsilon_{11}}{\partial K_{212}}-2\sigma_{12}\frac{\partial \varepsilon_{12}}{\partial K_{212}}-\sigma_{22}\frac{\partial \varepsilon_{22}}{\partial K_{212}})dV_{y}=0,\\ \left(2,2,1\right):\int_{Y}(\sigma_{22}y_{1}-\sigma_{11}\frac{\partial \varepsilon_{11}}{\partial K_{221}}-2\sigma_{12}\frac{\partial \varepsilon_{12}}{\partial K_{221}}-\sigma_{22}\frac{\partial \varepsilon_{22}}{\partial K_{221}})dV_{y}=0,\\ \left(2,2,2\right):\int_{Y}(\sigma_{22}y_{2}-\sigma_{11}\frac{\partial \varepsilon_{11}}{\partial K_{222}}-2\sigma_{12}\frac{\partial \varepsilon_{12}}{\partial K_{222}}-\sigma_{22}\frac{\partial \varepsilon_{22}}{\partial K_{222}})dV_{y}=0 \end{array}\right.$$

Integration of these two sets of partial differential equations for the strain delivers the expression of the three strain components:

(A5) $$\left\{\begin{array}{l} \varepsilon_{11}=E_{11}+K_{111}y_{1}+K_{112}y_{2}\equiv \frac{\partial u_{1}\left(y_{1},y_{2}\right)}{\partial y_{1}},\\ \varepsilon_{22}=E_{22}+K_{221}y_{1}+K_{222}y_{2}\equiv \frac{\partial u_{2}\left(y_{1},y_{2}\right)}{\partial y_{2}},\\ \varepsilon_{12}=E_{21}+\frac{1}{2}\left(K_{121}+K_{211}\right)y_{1}+\frac{1}{2}\left(K_{122}+K_{212}\right)y_{2}\equiv \frac{1}{2}\left(\frac{\partial u_{1}\left(y_{1},y_{2}\right)}{\partial y_{2}}+\frac{\partial u_{2}\left(y_{1},y_{2}\right)}{\partial y_{1}}\right) \end{array}\right.$$

Integration of the two first equations in (A5) deliver the two displacement components up to two integration functions $\left(y_{1}\right),g\left(y_{2}\right)$:

(A6) $$\left\{\begin{array}{l} u_{1}=E_{11}y_{1}+K_{111}\frac{{y_{1}}^{2}}{2}+K_{112}y_{1}y_{2}+g(y_{2}),\\ u_{2}=E_{22}y_{2}+K_{221}y_{1}y_{2}+K_{222}\frac{{y_{2}}^{2}}{2}+f(y_{1}) \end{array}\right.$$

The two unknown integration functions are determined by inserting the obtained displacement in Equation (A4) into the last relation in Equation (A5), yielding after isolation of the terms depending on each spatial coordinate two ODE’s for $f\left(y_{1}\right),g\left(y_{2}\right)$ that can easily be integrated to yield:

(A7) $$\left\{\begin{array}{l} f\left(y_{1}\right)=u_{02}-Ky_{1}+E_{12}y_{1}-K_{112}\frac{{y_{1}}^{2}}{2}+\left(K_{121}+K_{211}\right)\frac{{y_{1}}^{2}}{2}\\ g(y_{2})=u_{01}+Ky_{2}+E_{12}y_{2}-K_{221}\frac{{y_{2}}^{2}}{2}+\left(K_{122}+K_{212}\right)\frac{{y_{2}}^{2}}{2} \end{array}\right.$$

This deliver the final expression of the microscopic displacement versus the macroscopic strain and strain gradient tensors corresponding to the exact displacement field for a pure strain gradient continuum:

(A8) $$u^{\mathrm{hom}}\left(y\right)=\left|\begin{array}{l} {u^{\mathrm{hom}}}_{1}=u_{01}+Ky_{2}+E_{11}y_{1}+E_{12}y_{2}+K_{111}\frac{{y_{1}}^{2}}{2}+K_{112}y_{1}y_{2}-K_{221}\frac{{y_{2}}^{2}}{2}+\left(K_{122}+K_{212}\right)\frac{{y_{2}}^{2}}{2},\\ {u^{\mathrm{hom}}}_{2}=u_{02}-Ky_{1}+E_{21}y_{1}+E_{22}y_{2}-K_{112}\frac{{y_{1}}^{2}}{2}+\left(K_{121}+K_{211}\right)\frac{{y_{1}}^{2}}{2}+K_{221}y_{1}y_{2}+K_{222}\frac{{y_{2}}^{2}}{2} \end{array}\right.$$

in which constant **K** plays the role of a rigid body rotation, and the vector $u_{0}\left(x\right)=(u_{01},\;u_{02})$ describes a rigid body motion. Note that the macroscopic position **X** plays the role of a mere parameter here.
